# Screening and Preliminary Identification of *Asparagus officinalis* Varieties under Low-Temperature Stress

**DOI:** 10.3390/genes15040486

**Published:** 2024-04-12

**Authors:** Youju Ye, Shuangshuang Wen, Jiali Ying, Yunfei Cai, Renjuan Qian

**Affiliations:** Zhejiang Institute of Subtropical Crops, Wenzhou 325005, China; yeyj@njfu.edu.cn (Y.Y.); 15501131025@163.com (S.W.); 18805158018@163.com (J.Y.); cai41777@126.com (Y.C.)

**Keywords:** *Asparagus officinalis*, low temperature, *AoMYB56*, ABA

## Abstract

To meet the large demand for *Asparagus officinalis* in the spring market and improve the economic benefits of cultivating asparagus, we explored the molecular mechanism underlying the response of *A. officinalis* to low temperature. First, “Fengdao No. 1” was screened out under low-temperature treatment. Then, the transcriptome sequencing and hormone detection of “Fengdao No. 1” and “Grande” (control) were performed. Transcriptome sequencing resulted in screening out key candidate genes, while hormone analysis indicated that ABA was important for the response to low temperature. The combined analysis indicated that the *AoMYB56* gene may regulate ABA in *A. officinalis* under low temperature. And the phylogenetic tree was constructed, and subcellular localisation was performed. From these results, we speculated that the *AoMYB56* gene may regulate ABA in *A. officinalis*. The results of this research provide a theoretical basis for the further exploration of low-temperature response in *A. officinalis*.

## 1. Introduction

*Asparagus officinalis* (*A*. *officinalis*) is a plant in the Asparagaceae family that is native to the Mediterranean coast and Asia Minor and has been cultivated in China for more than 100 years [1]. The tender young stems of *A. officinalis* are suitable for consumption and rich in nutrition; they have a delicate aroma and crisp taste; and they can be consumed in cold dishes, fried food, soup, or processed into canned food. The regular consumption of asparagus can increase appetite and prevent hypertension and heart disease, among other effects. *A. officinalis* has high nutritional value and is a popular product in the vegetable market that is favoured by consumers, enjoying its reputation as the “king of vegetables” [2].

In plants, low temperatures influence their viability, even leading to plant death in severe cases [3,4]. Low temperature has a significant impact on plants originating in subtropical and tropical areas. The tolerance level of different plants to low temperatures usually varies among plant species [5,6]. Under low temperatures, once the plants receive the signal, their defence mechanisms are activated [7]. The mechanism of plant response to low-temperature stress has been gradually clarified under the development of molecular biology technology [8,9,10].

Endogenous hormone ABA can improve the abiotic stress (such as low temperature, drought, and salt) resistance of plants, which has been confirmed in various plants [11,12,13]. Under stress, ABA begins the response mechanism in plants through stress signal transduction, participates in the regulation of plant metabolism, alleviates plant stress damage, and improves plant stress resistance [14,15,16]). Moreover, ABA is an important promoter of low temperature resistance-related gene expression in plants [17,18,19]. ABA treatment can improve the low-temperature resistance of *Arabidopsis* [20], wheat [21], strawberry [22], *Gladiolus* [23], and other plants.

The *MYB* family is widely distributed in plants, mainly through the specific binding with cis-factors to activate or inhibit gene transcription process, and then involved in the regulation of plant growth [24]. Many plants have been found to include the *MYB* family, such as *A. thaliana* [25], *Brassica* [26], and so on [27,28]. Responses to various abiotic, the *MYB* family is also a vital regulatory factor, regulating plant stresses. In cotton, *GbMYB5* was a positive regulatory factor in response to drought tolerance [29]. In *Carthamus tinctorius* (*C. tinctorius*), the transcription level of *CtMYB63* in grape is significantly up-regulated under various abiotic stress conditions, and can enhance cold and drought tolerance by activating rapid anthocyanin synthesis [30]. In addition, previous reports showed that the MYB family could influence plant development by regulating ABA under cold stress. Masrur found that *AtMYBR1* can delay leaf senescence by regulating ABA synthesis in *Arabidopsis* [31]. In strawberries, R2R3-MYB *FaMYB10* response to ABA induction was found to promote strawberry pigment synthesis and fruit ripening [32].

This research aimed to provide insights for meeting the large demand for *A. officinalis* in the spring market and improving the economic benefits of cultivating asparagus. In *A. officinalis*, to explore the mechanism of molecular response to low temperatures, we first screened out “Fengdao No. 1” under low-temperature treatment. Transcriptome analysis revealed key candidate genes, while hormone analysis indicated that ABA is important for the response to low temperature. In asparagus, the results showed that *AoMYB56* regulates ABA in response to low temperature. This research provides a basis for the further exploration of the low-temperature response of asparagus.

## 2. Plant Materials and Methods

### 2.1. Plant Materials and Low-Temperature Stress Treatment

Six asparagus varieties (“Fengdao No. 1”, “Fengdao No. 2”, “Grande”, “Wenlv No. 1”, “Hangyu No. 6”, and “Jingang 111”), which were cultivated in the Zhejiang Institute of Subtropical Crops Herbarium (120′63 E, 27.99 E), were placed in the dark at 7 °C for 8 h and light at 15 °C for 16 h (75% humidity), for low-temperature tolerance testing in this study. Then, we observed the growth of the six asparagus varieties under low-temperature stress treatment.

### 2.2. Physiological Index Detection

The tips of “Fengdao No. 1” (repeat sample names: FM1, FM2, and FM3) and “Grande” (repeat sample names: GM1, GM2, and GM3) were chosen for further study. To test the superoxide dismutase (SOD) and peroxidase (POD) activities in the *A. officinalis*, 0.1 mg FM1, FM2, FM3, GM1, GM2, and GM3 were selected. Then, 1 mL SOD/POD extraction liquid was added. All samples were placed in ice bath homogenization. Then, they were centrifuged at 8000× *g* at 4 °C for 10 min, and the supernatant was removed and put on ice to be measured. The kit was obtained from Nanjing Jiancheng Bioengineering Institute (Nanjing, China). All measurements were repeated in triplicate.

### 2.3. Transcriptome Sequencing

The RNeasy Plant Mini Kit (Tiangen, Beijing, China) was used to extract the total RNA of the six samples (FM1, FM2, FM3, GM1, GM2, and GM3) [33]. The mRNA was enriched using magnetic beads with oligo (dT) after the sample quality was checked and was randomly disrupted with fragmentation buffer. First, the cDNA strand was synthesized using six-base random hexamers as templates. Second, the cDNA chain was synthesized after adding buffer, dNTPs, RNase H, and DNA polymerase. The cDNA was purified using AMPure XP beads, end-repaired, and A-tailed, and the adaptors were connected [34]. Agilent 2100 (Agilent, Beijing, China) was used to test the cDNA library.

### 2.4. Analysis of Transcriptomic Results

The asparagus genome (reference genome version: GCF_001876935.1_Aspof. V1) was used as a reference sequence for alignment and transcriptomic reconstruction. HISAT2-2.2.1, which was designed and developed by Hopkins University in the United States, is a fast and sensitive comparison software based on the BWT algorithm, and its core was developed on the basis of Bowtie2. Compared with tophat2, it has greater efficiency and provides more accurate results, and it has been widely used for the comparison of RNA-seq data. After the mapped data were obtained from the clean data and the asparagus reference genome was aligned, the insertion fragment length test and randomness test were used to evaluated the sequencing library quality.

The mapped reads’ number in each sample and the length of the transcripts were normalized using the FPKM calculation method, and the expression levels for each sample were obtained. DESeq (R package 4.1.1) was used to analyse differential expression among different samples. The significance (*P*) obtained with the original hypothesis test was corrected using the Benjamini-Hochberg correction method, and the Q value was obtained using the false discovery rate (FDR) of the differentially expressed genes (DEGs). KEGG metabolic pathway analysis and differential gene expression analysis were performed on the transcripts.

### 2.5. DEGs Identified Using qRT-PCR in A. officinalis

Eight DEGs were identified using qRT-PCR, while Oligo 7 software was used to design primers (Table S1). The total RNA of the tips in “Fengdao No. 1” and “Grande” was reverse-transcribed to synthesize first-strand cDNAs, which would be used in the qRT-PCR experiment programme. Then, 2 μL of cDNA, 10 mL of SYBR Premix Ex Taq, 3 μL of 50× ROX Reference Dye II, 0.2 μL (10 μM) of each primer, and 4.6 μL of H_2_O were used for a 20 μL mixture. ABI Viia 7 system (Applied Biosystems, Waltham, MA, USA) was used to conduct the experiment according to the manufacturers’ protocols [35], while three technical replicates were performed for each sample. Reference gene *18S* was used as a control for normalization. Relative expression levels in different tissues were calculated using the 2^−ΔΔ^ CT method.

### 2.6. Determination of Endogenous Hormone Levels in A. officinalis

The tips of “Fengdao No. 1” and “Grande” were collected and stored at −80 °C for subsequent use. One millilitre of methanol/water/formic acid (15:4:1, *V*/*V*/*V*) was used to obtain extracts from 50 mg of seeds. Under a nitrogen gas stream, the combined extracts were evaporated to dryness, reconstituted in 100 μL of 80% methanol (*V*/*V*), and then filtered through a 0.22 μm filter for further LC-MS analysis. Then, the AB 6500+ QTRAP^®^ LC-MS/MS platform was used to detect ABA and GA. To ensure the accuracy of the experiment, three replicates of each assay were performed.

### 2.7. Combined Analysis of DEGs and Endogenous Hormones

A combined analysis of 8 DEGs and 14 endogenous hormones was performed, and a correlation network was constructed. The correlation network was performed using the OmicStudio tool at https://www.omicstudio.cn/tool with the default program (12 January 2024) [36].

### 2.8. Phylogenetic Analysis between the AoMYB56 and MYB Genes in A. thaliana

Sequences of the MYB proteins of *A*. *thaliana* were downloaded from PlantTFDB, a plant transcription factor database (planttfdb.cbi.pku.edu.cn, 18 Feburary 2024). To analyse the sequences, DNAMAN 6.0 software was used to align the *AoMYB56* and *MYB* genes in *A. thaliana*. A phylogenetic tree of the *AoMYB56* and *MYB* genes in *A. thaliana* was constructed via the NJ (neighbour-joining) method in MEGA X software.

### 2.9. Subcellular Localisation of AoMYB56

First, coding regions of the *AoMYB56* gene were inserted into the entry vector pCR8/GW/TOPO. And then, pBI121 (with a C-terminal HA-tag) was driven by the cauliflower mosaic virus (CaMV) 35S promoter. The constructed expression plasmid was transformed into Agrobacterium. The Agrobacterium clone (EHA105) with a transformed-expression plasmid was selected and cultured in 1 mL LB liquid medium containing corresponding antibiotics at 200 rpm at 28 °C for 24 h. Then, 1 mL of cultured Agrobacterium solution was transferred to 20 mL of LB medium containing the corresponding antibiotic, which contained 15 μM acetylsyringone (20 mL of LB with 3 μL of 100 mM AS). The mixture was cultured at 28 °C, 200 rpm to the logarithmic phase of Agrobacterium growth (OD600 = 0.5–0.6). Then, it was centrifuged at room temperature at 5000 rpm for 10 min to collect the bacteria, and the bacteria were suspended with dye solution (containing 10 mM MgCl_2_, 10 mM MES, 150 μM acetylsyringone, pH = 5.6) to OD600 = 1.0. They were then left at room temperature for 2~3 h. Two kinds of bacteria containing different plasmids were mixed in equal volume; a small opening was gently made on the back of the tobacco leaf with a 1 mL needle (taking care not to pierce it), and then a needle tube with the needle removed was used to absorb the bacterial solution and inject it into the leaf from the wound site. Water-stained areas of tobacco leaves were marked with a marker. The injected plants were placed in the dark for 12 h, and then cultured at around 21 °C for 2 days to observe whether the tobacco area injected with Agrobacterium was fluorescent, and the labelled areas of tobacco leaves were torn off. Then, cellular localization was observed using confocal microscopy (Carl Zeiss, Oberkochen, Germany) with a fluorescence microscope and an excitation light source system (Lumen Dynamic Connections). Protoplast transfection was repeated three times for experiment accuracy.

## 3. Results

### 3.1. Growth of Six Asparagus Varieties under Low-Temperature Conditions

**Figure 1:**
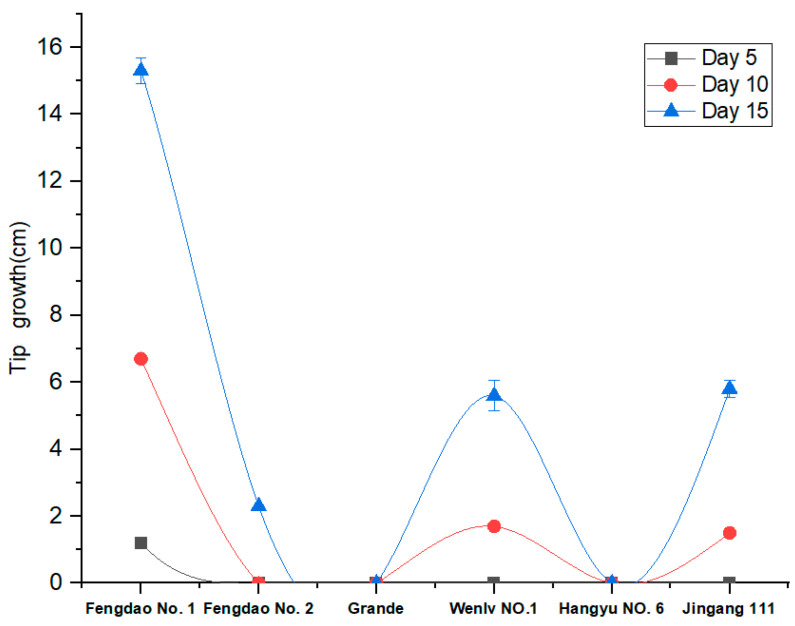
Growth conditions of six asparagus varieties under low-temperature, represented by a different colour. The X axis shows the low-temperature treatment time, while the Y axis shows the tip growth status.

The growth of six asparagus varieties (“Fengdao No. 1”, “Fengdao No. 2”, “Grande”, “Wenlv No. 1”, “Hangyu No. 6”, and “Jingang 111”) under low-temperature conditions was evaluated. The results indicated that only “Fengdao No. 1” grew by day 5, while “Wenlv No. 1” and “Jingang 111” grew by day 10. “Grande” and “Hangyu NO. 6” did not grow at low temperatures. Therefore, we selected “Fengdao No. 1” as the experimental material, and “Grande”, which grows at 25 °C, as the control material. The repeat samples “Fengdao No. 1” and “Grande” were named “FM1, FM2, FM3”, and “GM1, GM2, GM3” (Figure 1).

### 3.2. POD and SOD Activity Determination in “Fengdao No. 1” and “Grande”

**Figure 2:**
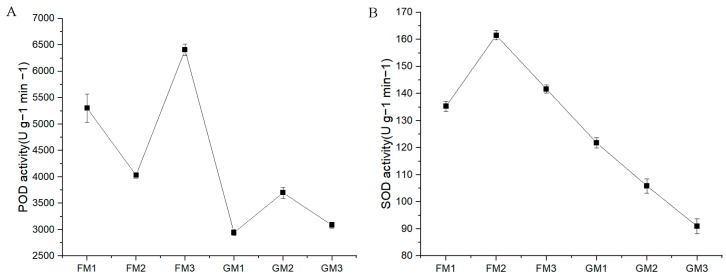
POD (**A**) and SOD (**B**) activity determination in “Fengdao No. 1” and “Grande”. (**A**) The X axis shows the different samples, while the Y axis shows POD activity. (**B**) The Xaxis shows the different samples, while the Y axis shows SOD activity.

The detection results indicated that POD activity was greater in FM than in GM. POD activity was highest in FM3 and lowest in GM1. Moreover, SOD activity was also greater in FM than in GM. SOD activity was highest in FM2 and lowest in GM3 (Figure 2).

### 3.3. Transcriptome Sequencing Data

Transcriptome sequencing was performed on the six samples of “Fengdao No. 1” produced at a low temperature and those of “Grande” produced at a normal temperature, and 39.80 Gb clean data were obtained. The clean data of each sample reached 5.76 Gb or more, GC content was greater than 45.31%, and the N base content (N) in the clean data was 0. The percentage of Q20 bases was more than 98.1%, and the percentage of Q30 bases was 95.16% or greater (Table 1).

**Figure 3:**
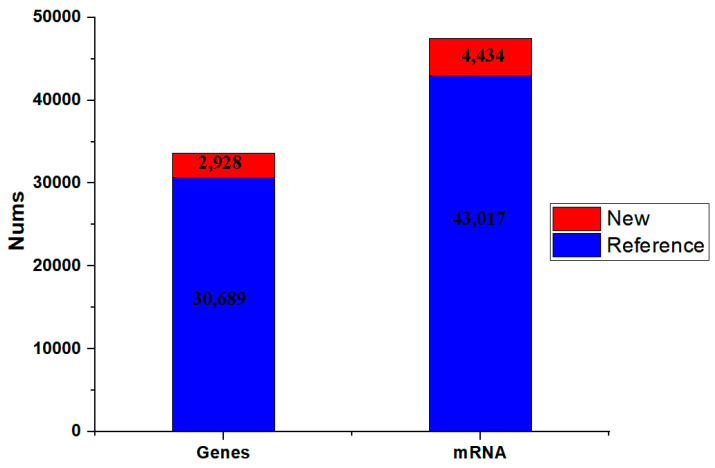
Transcriptome reconstruction results. The blue bar chart represents the number of genes compared with the reference genome, and the red bar chart represents the number of genes which are new transcript sequences. The X axis represents the numbers (Nums), and the Y axis represents the genes or mRNAs.

The published asparagus genome sequence was subsequently used as a reference sequence for alignment and transcriptome reconstruction. The clean reads of each sample were compared with the specified reference genome, and the efficiency of the comparison was greater than 90.39%. After StringTie was used to assemble the transcripts, 43,017 transcripts matched the reference genome, and 2709 transcripts were new transcripts. In addition, 30,689 genes could be compared with the reference genome, and the other 1957 genes were new transcript sequences, which may be related to differences between samples (Figure 3).

### 3.4. DEG Analysis between “Fengdao No. 1” and “Grande”

**Figure 4:**
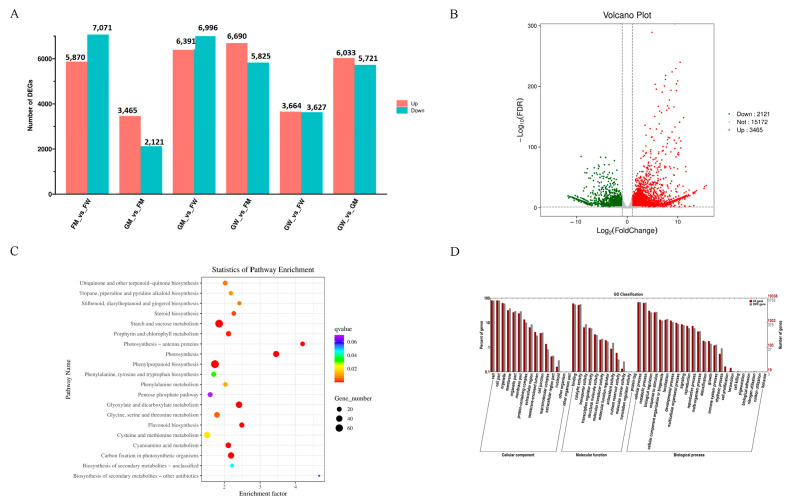
DEG analysis. (**A**) DEG annotations in the seven databases. The X axis shows the different databases, while the Y axis shows the gene number. Different databases are represented by different colours. (**B**) Volcano plot of DEGs. The X axis shows the FDR, while the Y axis shows the fold change. Green dots represent down-regulated genes, and red dots represent up-regulated genes. (**C**) KEGG pathway analysis of DEGs. The X axis shows the enrichment factor, while the Y axis shows the pathway name. The larger the circle, the more the genes. (**D**) GO enrichment of DEGs. The X axis shows the Go classification, while the Y axis shows the percentage of genes.

The DEG analysis revealed that 5488 DEGs exist between FM and GM (2072 up-regulated genes and 3416 down-regulated genes) (Figure 4A). Functional annotation was performed by comparing the DEGs with protein databases, and 3954, 3706, 1923, 1709, 4882, 2330, 3961, and 4878 genes were annotated with the Swiss-Prot, GO, KEGG, COG, KOG, Pfam, and NR databases, respectively (Figure 4B).

The result of KEGG enrichment revealed that most genes were enriched in the starch and sucrose metabolism pathway, followed by phenylpropanoid biosynthesis and carbon fixation in photosynthetic organisms (Figure 4C). The GO enrichment result indicated that most genes were enriched in the biological process (BP) category, followed by molecular function (MF) and cellular component (CC) (Figure 4D).

### 3.5. Candidate Key DEG Analysis and qRT-PCR Verification

**Figure 5:**
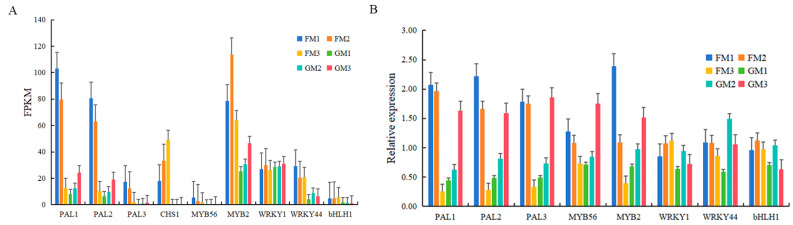
FPKM (**A**) and qRT-PCR (**B**) results of eight DEGs. Different samples are represented by different colours. The X axis represents the DEGs, while the Y axis represents FPKM (**A**) or relative expression (**B**).

To further study the function of key DEG response to low-temperature stress, we selected eight DEGs to perform the qRT-PCR experiment. Three DEGs belonging to the anthocyanin synthesis structural gene family, and another five DEGs belonging to the MYB-bHLH-WD40 (MBW) complex were selected. The results of qRT-PCR were consistent with the FPKM values, which indicated that the transcriptomic results were accurate and reliable (Figure 5).

### 3.6. Endogenous Hormone Analysis

**Figure 6:**
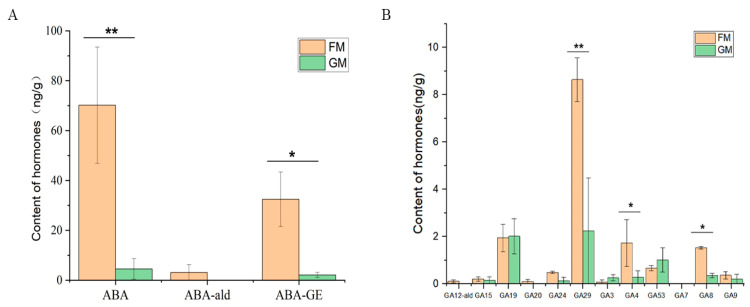
Endogenous hormone content in FM and GM. ABA-ald: abscisic acid aldosterone; ABA-GE: β-D-glucopyranosyl abscisate; GA12-ald: gibberellin 12 aldosterone. The X axis represents the different endogenous hormones, while the Y axis shows the hormone content. * represent significant (*p* < 0.05), ** represent highly significant (*p* < 0.01).

The results of the endogenous hormone analysis revealed that three ABA class hormones (ABA, ABA-GE, and ABA-ald) and nine GA class hormones (GA20, GA9, GA3, GA15, GA53, GA12-ald, GA7, GA29, GA24, GA4, and GA19) were detected in the six samples. The ABA content was highest in the six samples, followed by the ABA-GE and GA29 content. It is worth noting that ABA showed a significant difference between FM and GM (Figure 6).

### 3.7. Correlation Analysis of DEGs and Hormones

**Figure 7:**
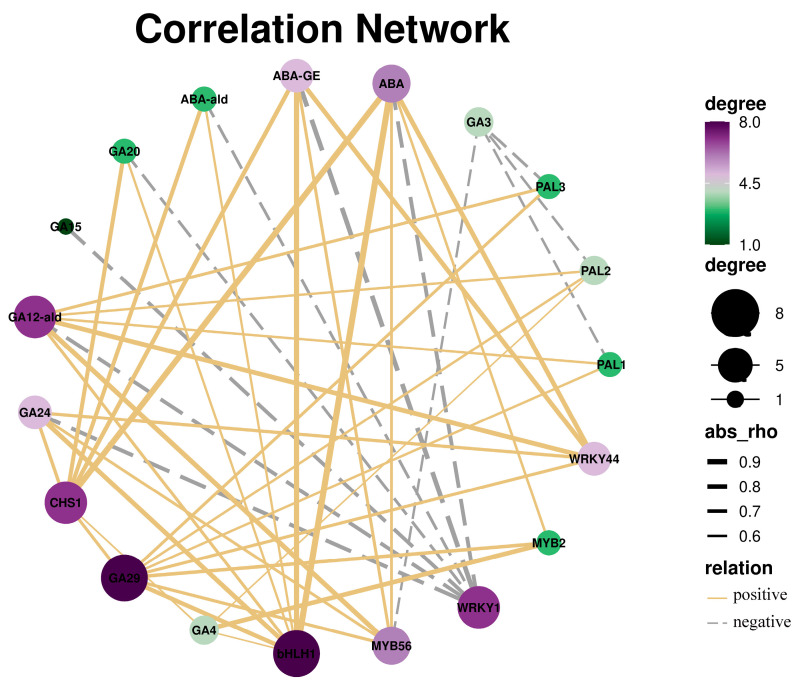
Correlation analysis of DEGs and hormones. Degree is represented by the colour and loop size. Positive is represented by the solid yellow line, while negative is represented by the grey dotted line.

The correlation analysis revealed that *bHLH1*, *CHS1*, and *MYB56* may be important genes that regulate ABA. The endogenous hormone detection result showed that the ABA content was significantly different between FM and GM. Therefore, we speculated that *MYB56* may be the key gene involved in the response to low temperature by regulating ABA in *A. officinalis* (Figure 7).

### 3.8. Preliminary Identification of AoMYB56

**Figure 8:**
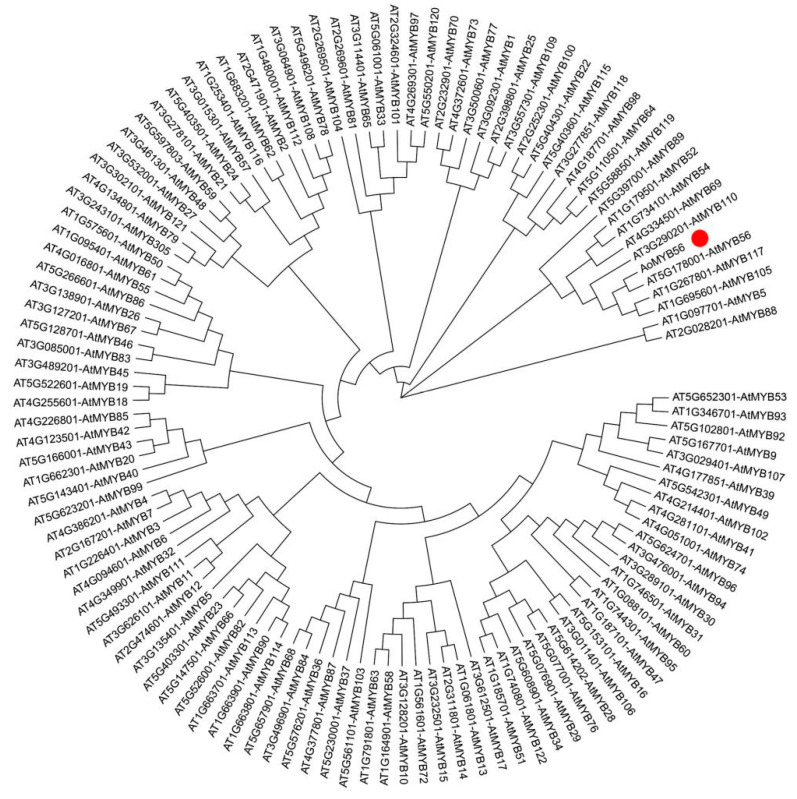
Phylogenetic tree of the AoMYB56 protein and MYB protein of *A. thaliana* constructed with the NJ (neighbor-joining) method in MEGA X.

An *evolutionary* tree was constructed using MEGA X (1000 bootstrap), to determine the relationships between the MYB protein of *A. thaliana* and the AoMYB56 protein. The *evolutionary* tree showed that AoMYB56 shared the highest homology with AtMYB56, followed by AtMYB117, AtMYB105, and AtMYB110 (Figure 8).

### 3.9. Subcellular Localization of AoMYB56

**Figure 9:**
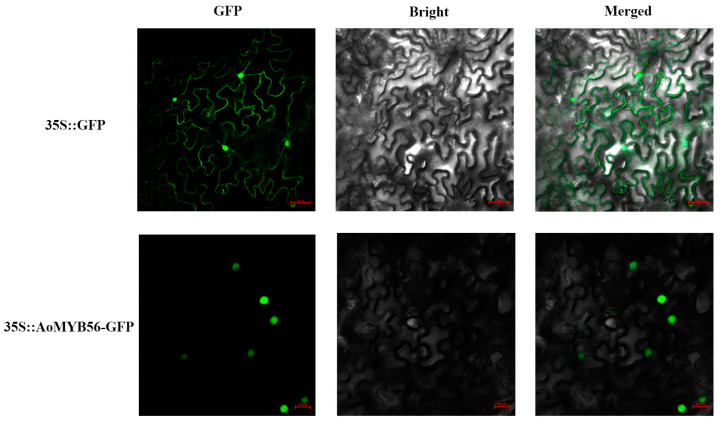
Subcellular localization analysis of the AoMYB56 protein. Merged: GFP+ Bright; GFP: green fluorescent protein. The control protein is represented by the 35S-GFP fusion. The red line represents a scale bar of 10 μm.

First, we used MLEG to identify AoMYB656 and found that it was localized in the nucleus. To verify this result, we constructed 35S::GFP-AoMYB56 plasmids and transfected them into tobacco protoplasts. Moreover, 35S::GFP fusion protein was expressed in the tobacco protoplast nucleus and cytomembrane as a positive control. The result of confocal microscopy showed that the plasmids were located in the nucleus, which is consistent with the predicted outcome (Figure 9).

## 4. Discussion

Low temperature is a vital environmental factor influencing plant growth, which delays the growth and development of plants, causing them to stagnate [37]. The production of *A. officinalis*, as an important vegetable worldwide, is often limited by low temperatures. Low temperatures slow the growth of *A. officinalis* and reduce its economic benefits. Therefore, it is important to identify low-temperature-resistant varieties of *A. officinalis* and explore the underlying molecular mechanism for further directive breeding to improve the economic benefits.

In this study, low-temperature-resistant varieties of *A. officinalis* were selected using a low-temperature treatment, and “Fengdao No. 1” was selected from six varieties. Transcriptome analysis between FM and GM was performed, and the results showed that 5488 DEGs were detected. Most DEGs were enriched in metabolic process categories. In our hormone detection experiment, “Fengdao No. 1” exhibited greater SOD and POD activities than the control group “Grande”. Previous study indicated that abiotic stress induces the accumulation of reactive oxygen species (ROS), while ROS-scavenging enzymes (SOD and POD) can protect plants from oxidative damage [38]. The result of our research indicated that the low-temperature tolerance of “Fengdao No. 1” may be enhanced by activating the ROS-scavenging enzyme system. That is probably because that the metabolic process system is the first to experience damage in plants under low temperatures, followed by a series of physiological and biochemical reactions.

ABA has been reported as an important hormone regulating abiotic stress in plants, including rice [39], maize [40], banana [41], and many other plants [42,43,44]. In lily, Cai found that Fluridone, a synthetic inhibitor of ABA, can effectively prevent the occurrence of dormancy in a lily tissue culture at low temperatures [45]. In this study, the ABA content was greater in “Fengdao No. 1” than in “Grande”. *A. officinalis*, like lily, is a bulbiferous plant; therefore, we speculated that ABA could promote development in “Fengdao No. 1” under low temperatures.

Evolutionary tree analysis showed that *AoMYB56* shared the highest homology with *AtMYB56*. In a previous study, Zhang and colleagues reported that *AtMYB56* could promote seed growth [46]. Therefore, we speculated that *AoMYB56* may have a function similar to *AtMYB56*. The correlation analysis indicated that ABA was regulated by *AoMYB56* in this study. Therefore, we speculated that *AoMYB56* maybe the transcriptional activator of ABA signalling in *A. officinalis*. A previous report indicated that *AtMYC2* (*bHLH*) and *AtMYB2* are transcriptional activators of ABA signalling [47]. Plant transcription factors play important roles in low-temperature responses, and numerous *MYB* genes have been shown to respond to low-temperature stress [48,49]. Combining the above results, this study showed that *AoMYB56* may act as a positive regulator under low-temperature stress by regulating ABA. This finding is consistent with previous reports showing that *MYB* is frequently involved in the ABA signalling-mediated regulation of plant adversity responses [50,51,52]. However, uncovering the detailed function of *AoMYB56* requires further research, which we will conduct in future work.

## 5. Conclusions

Low temperatures influence the growth and development in almost all plants, include the important vegetable *A. officinalis*. To meet the large demand for *A. officinalis* in the spring market and improve the economic benefits of cultivating asparagus, the molecular mechanism underlying the response of *A. officinalis* to low temperatures was explored in this research. “Fengdao No. 1” was selected as a low-temperature-tolerant variety, and the transcriptome analysis and hormone analysis indicated that *AoMYB56* may regulate ABA in *A. officinalis*. This research provides a theoretical basis for the further exploration of the low-temperature response in *A. officinalis*.

## Figures and Tables

**Table 1:** Transcriptome sequencing data in *A. officinalis*.

Sample	ReadSum	BaseSum	GC (%)	N (%)	Q20 (%)	Q30 (%)
FM1	23,817,457	7,145,237,100	45.35	0	98.25	94.73
FM2	19,199,653	5,759,895,900	46.01	0	98.24	94.74
FM3	20,242,141	6,072,642,300	45.31	0	98.1	94.4
GM1	22,487,796	6,746,338,800	45.89	0	98.26	94.66
GM2	22,890,147	6,867,044,100	46.61	0	98.4	95.16
GM3	24,023,866	7,207,159,800	45.76	0	98.28	94.8

## Data Availability

The data were uploaded to the NCBI database (https://www.ncbi.nlm.nih.gov/Traces/study/?acc=PRJNA1049888, accessed on 2 November 2023). The data that support the findings of this study are available from the corresponding author upon reasonable request.

## Supplementary Materials

The following supporting information can be downloaded at: https://www.mdpi.com/article/10.3390/genes15040486/s1, Table S1: The primers of qRT-PCR.
